# Information and Communication Technology-Based Application for Cognitive Behavioral Therapy among Community-Dwelling Older Adults with Insomnia: Development and Validation Study

**DOI:** 10.3390/healthcare12010106

**Published:** 2024-01-02

**Authors:** Yeonhee Lee, Inseong Kim, Seonheui Lee, Soyoung Yu

**Affiliations:** 1 Department of Nursing Science, College of Nursing, Gachon University, Incheon 21936, Republic of Korea; sm200702@smgh.co.kr; 2 Health Insurance Review & Assessment Service, Wonju-si 26465, Republic of Korea; mjkis3325@hira.or.kr; 3 College of Nursing, CHA University, Pocheon 11160, Republic of Korea

**Keywords:** community-dwelling older adults, cognitive behavioral therapy, information and communication technology, insomnia, mobile app

## Abstract

This study developed an information and communication technology-based mobile application to administer cognitive behavioral therapy to community-dwelling older adults with insomnia. First, the content of the application was determined through a systematic review and preference survey. Preference data on the perception, needs, and preference for non-face-to-face service content were collected from 15 July 2021 to 31 August 2021 from 100 community-dwelling older adults aged 65 years and older. In the design stage, the structure and function of the application were determined, and an interface was designed. The application was developed in conjunction with design experts and programmers using Android Studio software (Android 9). Usability tests were conducted during the implementation stage, followed by an evaluation stage. The evaluation revealed that the application’s structure and functions should comprise sleep information, sleep-habit improvement, sleep assistance, video, real-time counseling, and exercise services. These elements were finalized after receiving the results of a preference analysis and advice from an advisory panel of experts in different fields. The developed application was rated with a score of four or higher in all areas. This study successfully developed, implemented, and evaluated a new mobile application called Smart Sleep for community-dwelling older adults with insomnia.

## 1. Introduction

Sleep-related problems are common among older adults; it has been reported that 40–70% of older adults experience chronic sleep problems, and up to 50% remain undiagnosed [1]. Sleep disorders associated with aging are characterized by a decrease in total sleep time, difficulty initiating sleep, early waking, a decrease in deep sleep (i.e., slow-wave sleep and REM sleep), fragmented sleep due to frequent waking at night, and the need for daytime naps [2]. Altered sleep–wake patterns among older adults are caused by decreases in slow-wave sleep, which, in turn, is related to aging and the associated decrease in sleep efficiency. Poor sleep efficiency means that older adults experience frequent awakening, resulting in excessive daytime sleepiness, memory loss, poor concentration, and/or a lower quality of life [3]. In 2017, the American Academy of Sleep Medicine and the European Society of Sleep Medicine published guidelines for the treatment of insomnia [4,5]. These guidelines strongly recommend non-pharmacological treatments, especially cognitive behavioral therapy (CBT), for insomnia (CBT-I) and suggest that medical treatments be administered only when CBT-I proves to be ineffective or when it is impossible to administer [6]. Owing to physical and psychological dependence, older-adult patients with sleep disorders who have used pharmacological sleep aids for a long time may find it difficult to stop taking the medication; therefore, non-pharmacological management is a better and more effective method for treating chronic sleep disorders [7]. Among the non-pharmacological methods for managing insomnia, experts predominantly recommend CBT-I [8]. CBT-I comprises an integrated intervention that includes sleep restriction therapy, relaxation therapy, sleep hygiene education, and stimulation control therapy [9]. A meta-analysis recently demonstrated the effectiveness of CBT-I for older adults with sleep disorders through a systematic literature review [7,10,11].

Since the onset of the coronavirus disease (COVID-19) pandemic, individual economic activities, face-to-face treatments, and long-distance travel have been affected. However, developments in information and communication technology (ICT) have enabled newer means of providing appropriate healthcare for older adults [10,12]. Consequently, considering individuals’ aggravated financial and physical situations in the wake of the pandemic, the need for telemedicine and mobile health has increased [13]. Therefore, a smart healthcare system for older adults should be established and actively used. This system would also facilitate a reduction in the social costs of health promotion among older adults [14]. Additionally, the use of AutoML could reveal the challenges and limitations of using the aforementioned tools on medical data and may serve as a basis for future improvements to better solve medical problems [15]. In terms of using ICT to improve the discomfort experienced when visiting medical institutions, one study showed that 66% of the respondents were satisfied with ICT-based services, while 31% reported being moderately satisfied with ICT-based services. Therefore, it can be said that most participants were satisfied with the accessibility and convenience offered by ICT-based services [16].

ICT-based applications can be used by older adults for various purposes. When such applications are used indirectly for other tasks such as direct interactions with people, they improve the quality of life of older adults by facilitating their participation in society [17,18]. Regarding healthcare provision to older adults, it has been observed that ICT-based applications improve their quality of life, reduce depression, offer psychological stability, and improve family and social relationships [19,20,21,22,23].

It has also been reported that non-face-to-face CBT-I can be effective in treating older adults [24]. In a study on the feasibility and acceptability of administering CBT-I to patients by developing a mobile phone application, it was observed that the application did not impair or weaken the benefits of CBT-I, leading to significant improvements in participants’ sleep outcomes [25]. The same study found that patients consistently utilized the application as prescribed, specifically engaging with the sleep diary and notification functions over a six-week period, resulting in notable enhancements in sleep outcomes. Another study reported a significant change in insomnia severity index (ISI) scores among US soldiers with sleep disorders who have served in Iraq or Afghanistan since 2001, indicating reduced insomnia symptoms after the treatment [26].

Several studies have focused on ICT-based healthcare applications. One systematic review and meta-analysis investigated the effects of computer-based CBT (CCBT-I) on individuals older than 18 years and found that CCBT-I had a beneficial effect in the treatment of insomnia [27]. Another systematic review and meta-analysis confirmed that ICT intervention positively affected sleep disorders among older adults, depression, and quality of life, as well as indicators directly related to sleep, such as ISI and sleep quality [28].

In terms of existing research on ICT-based interventions, there are currently very few applications that use evidence-based principles to practice behavioral and cognitive skills to help users manage insomnia [29]. Moreover, although the use of CBT-I mobile applications has recently attracted the attention of mental health experts and researchers, it remains to be determined whether this application is effective for older users [30]. 

Nevertheless, some studies have also proposed the necessity of sleep applications, suggesting that CBT-I Coach applications can improve subjective sleep [26]. However, the absence of easily accessible CBT applications for insomnia in older adults emphasizes the importance of sleep applications. This tool has the potential to improve sleep quality and overall quality of life in older individuals with insomnia. Therefore, this study aimed to develop an ICT-based application for administering CBT to community-dwelling older adults with insomnia and evaluate the validity of the application.

## 2. Materials and Methods

This study developed an ICT-based application to administer CBT to community-dwelling older adults with insomnia using a mixed-methods research design. After development, experts evaluated the content validity according to the ADDIE model [31,32,33]. The model follows five steps: analysis, which is the process of clarifying what should be done and identifying requirements; design, which includes specifying goals and appropriate learning materials and methods; development, which involves creating specific textbooks for participants; implementation, which includes executing what has been designed and developed directly in a user environment; and evaluation, which relates to refining and complementing the designed program using various assessments [34]. The ADDIE model consists of distinct stages. To prevent confusion, a brief overview of the general outcomes and connections between each phase is presented.

### 2.1. Step 1: Analysis 

Participants: The ICT-based application development content was formulated based on data from a systematic review and preference survey. Literature searches included Ovid Medline (1946–June 2021), Ovid EMBASE (1974–June 2021), and the Cochrane Library. Posters, abstracts, animal research, and publications in languages other than English or Korean were excluded following specified criteria. Two reviewers independently assessed the titles and abstracts, and one independently extracted the data. Population characteristics for the ICT intervention, including age, sex, medical history, and ISI scores, were collected. The analysis considered 16 studies (10 RCTs, 5 quasi-experimental studies, and 1 retrospective study) using the RoB tool, revealing a low attrition risk and reporting bias. Most studies were from the US (10) and predominantly published in 2019 and 2020 [28]. 

Keywords related to gerontology, ICT intervention, and sleep disorders guided the study, encompassing terms such as aged, older, elderly, mobile application, telemedicine, and sleep disorders. After evaluating 47 studies, 31 were excluded for reasons such as irrelevant patients or interventions, gray literature, duplicate studies, or one systematic review. Ultimately, 16 studies were selected for analysis and the components necessary for ICT intervention were identified. Data included the inclusion/exclusion criteria, and a meta-analysis revealed a significant reduction in ISI in the CBT-I group from six RCTs and four quasi-experimental studies. 

A preference survey questionnaire was developed based on the advice of three nursing professors, a public health doctor, a neuropsychiatrist, and three geriatric nurses. The content validity of the questionnaire was evaluated. A preference analysis was performed on 100 community-dwelling adults aged 65 years or older from 15 July to 31 August 2021. Written informed consent was obtained from all the participants. Participants with insomnia were surveyed using a questionnaire. The main contents of the survey were as follows: whether the participants had experience with insomnia, whether they had experience using other insomnia applications, whether they had experience taking sleeping pills, whether there was a need to develop an insomnia application for older adults, and their preferences for the application content, general characteristics, and non-face-to-face service requirements. Needs and preferences were developed based on a systematic review. The collected data were analyzed using IBM SPSS Statistics version 26 (IBM Inc., Armonk, NY, USA). The participants’ general characteristics and preferences were analyzed by calculating frequencies, percentages, averages, and standard deviations.

Results: After evaluating 47 studies and examining the full text of the relevant studies extracted during the literature review, 31 were excluded for the following reasons: 17 did not include relevant patients, 6 did not include relevant interventions, 3 were gray literature, 4 were duplicate studies, and 1 was a systematic review. Finally, 16 studies were included in the analysis. Based on the data obtained from these 16 studies, the components necessary for ICT interventions were identified. The collected data included inclusion and exclusion criteria such as age, sex, medical history, and ISI score. A meta-analysis of six RCTs found a significant reduction in ISI in the CBT-I group compared to the control group, and four quasi-experimental studies showed a significant reduction in ISI after intervention.

### 2.2. Step 2: Design

Procedures: The structure and function of the mobile application were determined based on data obtained during the analysis stage, and an interface was designed. The main menu of the designed application, “Smart Sleep”, comprises sleep information, sleep-habit improvement, real-time counseling and exercise, a sleep assistant, and videos. Sleep information included a sleep diary, a sleep activity record, and an ISI-based questionnaire. Sleep-habit improvement included completing a sleep questionnaire wherein the participants wrote their sleep-related details. For real-time counseling and exercise, real-time classes were conducted via online counseling, and real-time training was conducted with the manager. Exercise included participants performing walking exercises daily, and the videos included participants watching fall prevention exercises. To help users sleep, the application included meditation exercises, sleep music, and natural sounds. The video section included educational and relaxation training videos.

**Figure 1:**
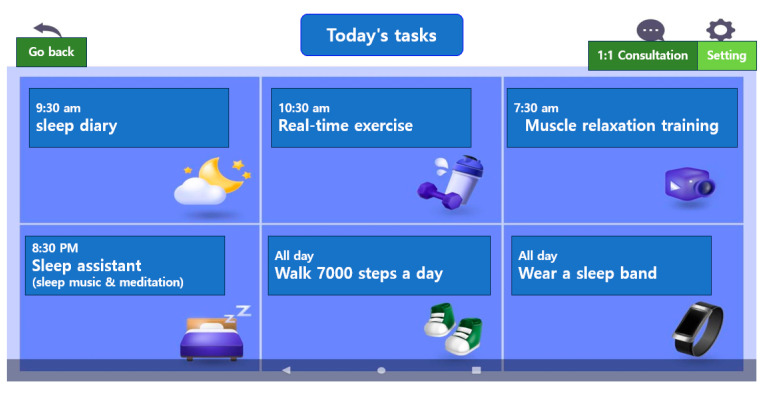
Today’s to-do contents and today’s to-do completion.

Results/Outcomes: All activity records of the participants were stored in the administrator’s server; when the participants did not execute the day’s task, it was possible to manually send them a notification to increase their level of participation. The interface included easy-to-read layouts, large texts, and intuitive pictures. Considering the participants’ ages, an icon explaining how to use the application content was created on the main screen and placed so that the participants could easily find and use it (Figure 1).

### 2.3. Step 3: Development

Participants: The researchers determined the structure and function of the mobile device application and designed the interface based on the data obtained during the analysis phase.

Procedures: To enable older adults to access the application easily, it was designed such that when the main content was tapped, participants could access the sub-content without any difficulties. The image materials used in the program were illustrated by the researcher and subsequently edited by the designer. They were either captured by the researcher or derived from free images, which were processed by both the researcher and the designer. The participants were notified of what they needed to do daily and incentivized to ensure participation. Appropriate feedback was provided through counseling and education.

**Figure 2:**
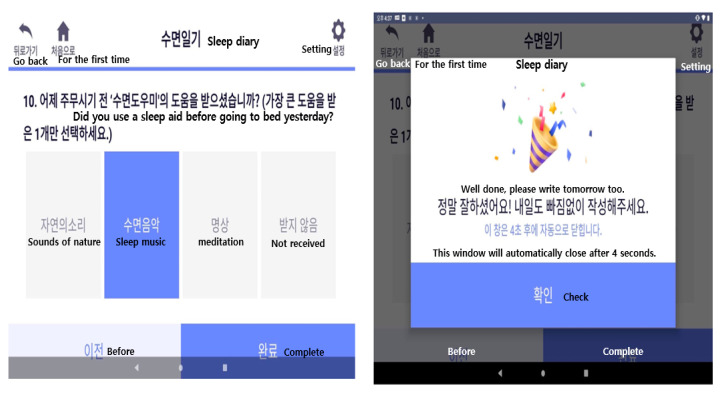
Sleep diary screen and sleep diary completion screen.

Results/Outcomes: After the researchers confirmed the finished prototype, the developers and designers made modifications, incorporating supplements (Figure 2).

### 2.4. Step 4: Implementation 

Participants: During the implementation stage, seven experts, including two elderly nurses, two nursing professors, two program experts, and one medical expert, participated in a usability test.

Procedure: Experts installed the mobile application on a tablet PC, and the researchers verified its validity. Information and participation instructions were provided to the elderly receiving care at senior welfare and public health centers, and 10 people were randomly selected from those who volunteered to participate.

Results: Each expert group had an Android-based tablet PC (Lenovo, 10.1, 1920 × 1200) equipped with Smart Sleep for seven days, wore a sleep band, kept a sleep diary, used a sleep aid app, and watched a sleep video. 

As with the expert group, 10 elderly participants used an Android-based tablet PC (Lenovo, 10.1, 1920 × 1200) equipped with Smart Sleep at home for seven days. On the first day, the researcher directly instructed the participants on how to use Smart Sleep and provided them with a sleep band for seven days. If older subjects had additional questions while using the app, they would call the researcher to resolve the issue; if necessary, the researcher would go home and meet them in person to resolve the issue.

### 2.5. Step 5: Evaluation

Participants: One week after using the application, the seven experts rated it using the Mobile Application Rating Scale (MARS) [35]. After one week of using the application, the 10 users evaluated the application using the user version of MARS (uMARS). Procedure: Similar to MARS, uMARS comprises five subdomains. However, the Sleep Information domain consists of 4 items for a total of 20 items.

Procedure: The MARS for professionals consists of 23 questions in five subdomains: participation, functionality, aesthetics, information, and subjective quality. 

Results: Each item was rated on a 5-point Likert scale. The higher the score, the better the application usability.

### 2.6. Ethical Considerations

This study was approved by the Institutional Review Board (IRB) of Gachon University (IRB No. 1044396-202106-HR-126-01). While recruiting participants, the study’s purpose was explained, and data were collected only from participants who provided written informed consent. The participants’ wishes were strictly adhered to, and they were assured that the research data and their personal information would not be used for any purpose other than the research. They were also assured that the research data would be stored in a secure location with a lock and password and that the collected data would be discarded after the study was completed. 

## 3. Results

### 3.1. Cognitive Behavioral Therapy for Insomnia (CBT-I) 

In one RCT, the total sleep time of the CBT-I group was significantly improved compared to the control group, and the awakening time after sleep onset was significantly reduced in the CBT-I group compared to the control group. Additionally, the RCT found a significant reduction in depression in the CBT-I group compared to the control group. A systematic literature review confirmed that the quality-of-life mental component score showed significant improvement in the CBT-I group compared to the control group. CBT-I is a simple, multifaceted treatment approach that addresses the psychological and behavioral factors associated with perpetuating sleep disorders [36]. Among the elements that constitute CBT for sleep disorders, the behavioral elements include stimulus control therapy, sleep restriction therapy, and muscle relaxation therapy for sleep disorder; furthermore, the cognitive elements are similar to CBT for depression. Sleep hygiene includes education on common sense and general behavioral principles about sleep [6,8,28,37].

Sleep stimulation control and sleep restrictions were designed to keep the participants’ sleep-awareness schedule consistent, eliminate harmful behaviors in bed and bedroom, and limit the amount of time needed to fall asleep, creating effective and enhanced sleep patterns [38]. Stimulation control therapy aimed to create a consistent sleep–arousal pattern by changing the bed and bedroom environments to break the connection with sleep-disturbing behaviors and act as a conditioned stimulus linked to sleep-enhancing behavior [8]. For sleep restriction, the total bedtime was gradually reduced by 15 or 30 min weekly, referencing the user’s sleep diary, until 85% sleep efficiency was achieved, as per the Korean version of the clinical treatment guidelines for insomnia [39]. The sleep efficiency rate suitable for each individual was then computed based on the sleep diary, following the sleep efficiency calculation method outlined in the Korean version of the Clinical Treatment Guidelines. A program was developed to evaluate the participants’ daytime symptoms, such as fatigue, daytime sleepiness, and concentration, and to restrict sleep while checking whether the patients’ subjective symptoms were improving [39]. In this study, as part of sleep stimulation and sleep restrictions, the Smart Sleep application required participants to enter one week’s sleep habits every Sunday and check the state of the sleep habits to adhere to habits that stimulate sleep. In addition, the sleep habit questionnaire included necessary information for sleep hygiene, and the researcher checked the sleep habit questionnaire so that habits could be improved through real-time counseling.

**Figure 3:**
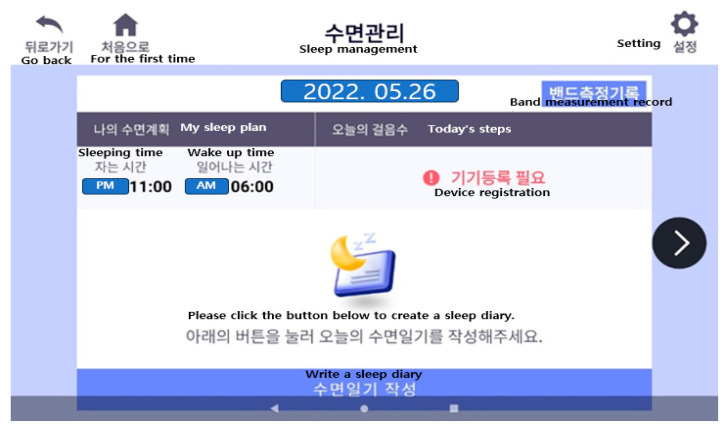
Sleep management (sleep restriction screen).

We used sleep restriction therapy to calculate sleep efficiency based on data from the patient’s sleep diary and sleep band and informed the participants of the ideal time to go to bed and wake up. Sleep diaries were written by participants every day and included the time they went to bed, the time it took to fall asleep, the time they wake up in the morning, and the time they get out of bed. Sleep hygiene included days in the past week when you only fell asleep when you felt sleepful, days when you only used your bed when you were sleeping, days when you got out of bed after being unable to sleep for 20–30 min, and days when you were unable to sleep. The application also helped the participants improve by determining the number of days they consumed caffeine (coffee, etc.) in the afternoon, the number of days they consumed alcohol, and the number of days they dimmed the lights while sleeping (Figure 3).

Relaxation therapy included muscle relaxation and abdominal breathing training videos. Muscle relaxation therapy is said to help patients with sleep disorders manage difficulty in going to sleep and increase the total sleep time [40].

The results of the preference analysis were as follows: Regarding the necessity of ICT non-face-to-face service, 77 people (77%) responded that they needed it, and the majority marked “yes” (72%), for the intention of using it. Their needs and preferences were determined through a systematic review. The participants preferred breathing exercises, stretching videos before going to sleep, meditating on music, and listening to natural sounds (Table 1). Based on the results of the preference analysis, an advisory group of experts was consulted regarding the content of the application composition plan. The experts included two nursing professors, two geriatric nurses, one psychiatrist, and one doctor of public health doctor. On the grounds of the consultation, after receiving advice on the necessity of sleep stimulation control, sleep restriction therapy, and sleep diaries, it was decided that these would be included in the application contents, despite ranking relatively low in preference in the preference survey. Consultation was conducted with the advisory group via focus group interviews and non-face-to-face video conferencing.

### 3.2. Results of the Application Design and Development

**Figure 4:**
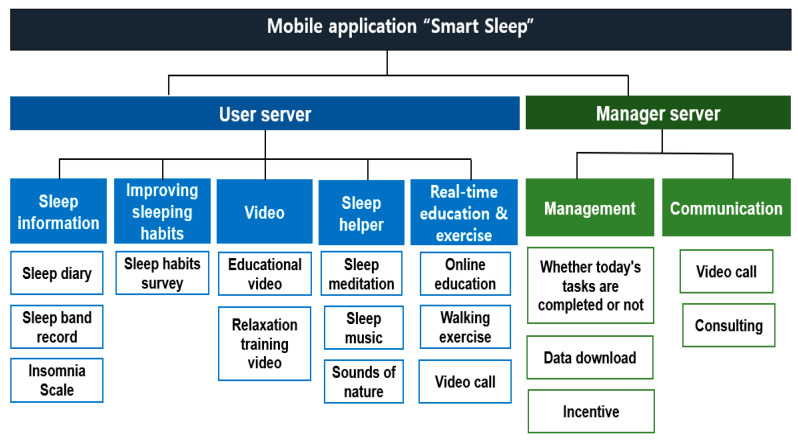
Information architecture of user and administrator server.

At the design stage, the structure and functions, including sleep information, sleep- habit improvement, sleep assistance, video, real-time counseling, and exercise services, were decided after receiving the preference analysis results and the advice from a panel of experts. In addition, to promote the execution ability of the application, a screen accessible to the administrator was added, and a configuration plan for feedback was included (Figure 4). 

**Figure 5:**
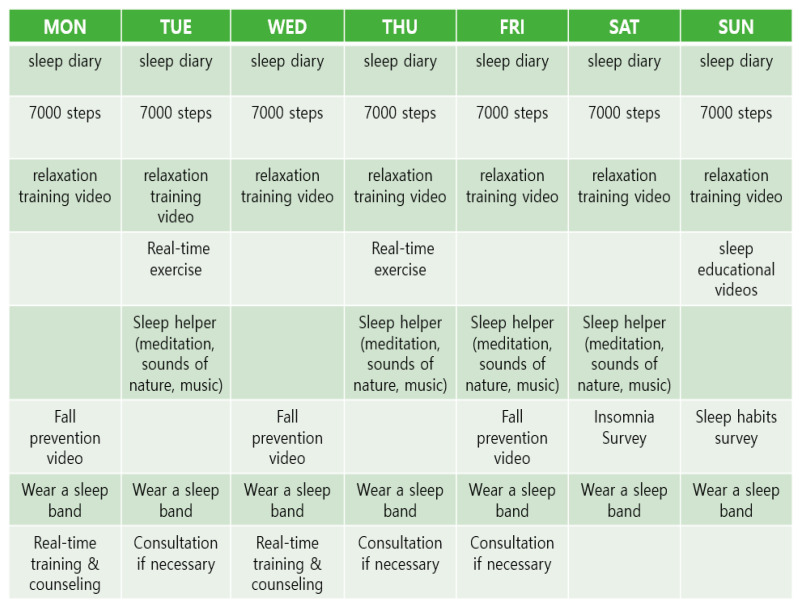
Smart Sleep program composition.

Sleep restriction, stimulus control therapy, relaxation training, cognitive strategies, and sleep hygiene education are representative cognitive behavioral treatments for insomnia that have been proven effective [37]. The Smart Sleep application also uses a sleep diary and a sleep band to obtain sleep efficiency information on what limits sleep. It includes relaxation training videos as well. During counseling and education, sleep hygiene education and information on sleeping habits were provided to help control stimulation (Figure 5).

Starting with a screen explaining how to write a sleep diary, users were asked to write a subjective evaluation of their sleep quality by entering the time they went to sleep and the time they awoke, for a total of 10 items. The manager was encouraged to write a sleep diary by providing the first regular notification every day and by being sent sent a push notification if it was incomplete.

Sleep activity was recorded using sleep-specific wearable bands worn by the participants. The sleep activity record was automatically uploaded to the manager’s screen daily and managed accordingly. The ISI questionnaire was prepared once every Saturday and consisted of seven questions. Participants were tasked with completing a sleep-habit improvement questionnaire once every Sunday. Regular notifications were given, and push notifications were sent to the administrator when tasks were not completed so that the survey could be managed without missing data.

Sleep therapy includes guided meditation, sleep music, and natural sounds. For convenience, a bar that allowed the user to select one of the four types immediately was installed at the top-right of the screen so that the participants could select them directly within the corresponding screen without going to the previous screen.

The videos included educational and relaxation-training videos. The educational videos provided information on sleep and insomnia, healthy sleep habits, insomnia treatment, breathing training, and educational relaxation training. Four educational videos were scheduled weekly. Relaxation training videos were made available daily before sleep.

Health measurements were set to walk more than 7000 steps, and the number of steps was measured using a wearable band and uploaded to the administration program.

Real-time exercise was conducted at a public health center, followed by gymnastics and walking for older adults, and users participated throughout the week. The administrator monitored the usage and input data.

**Figure 6:**
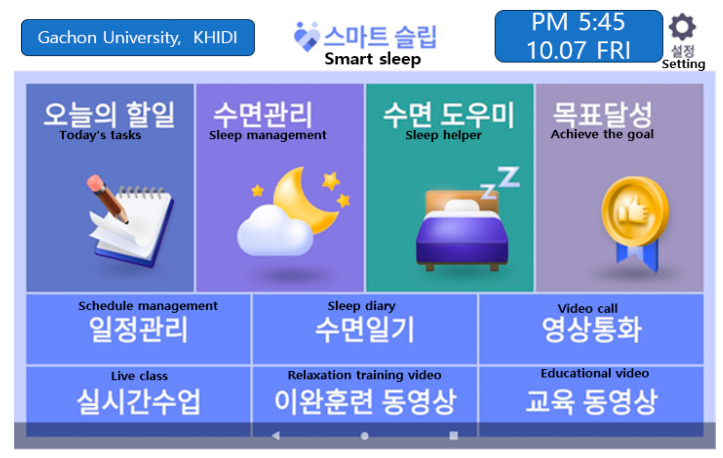
The interface of the mobile application “Smart Sleep”.

The administrator screen displays the current day’s to-do list and goal achievement. In this current scenario, the work is set for each day of the week, and if the achievement criteria are met, they are automatically marked as completed. There are six tasks in a day, and if a user completes five or more of these tasks, the user is considered to have completed the tasks. When a user performs five or more tasks, they can enter them into goal accomplishments and check whether they are “stamped”. On the administrator’s screen, if today’s to-do list is not implemented, the administrator sends a push notification to the participant (Figure 6).

### 3.3. Implementation Results and Evaluation: Usability Testing by Users and Experts

The Smart Sleep application was evaluated by seven experts, including two nursing professors, three geriatric nurses, one nurse, and one doctor of health science, using the MARS instrument in the review excerpts. The application was rated with a score of four or higher in all application programs. The mean score for support quality was 4.58 ± 0.31. For each category, subjective quality (4.37 ± 0.44), information (4.40 ± 0.37), function (4.35 ± 0.51), participation (4.78 ± 0.25), and aesthetics (5.00 ± 0.00) were the highest (Table 2).

Additionally, the uMARS was evaluated by 10 older adults who used the application. It was also evaluated with a score of four or higher across all categories. The mean score on the overall support quality assessment was 4.46 ± 0.42. Information (4.72 ± 0.35), aesthetics (4.56 ± 0.50), participation (4.45 ± 0.45), functionality (4.35 ± 0.36), and the application’s subjective quality (4.26 ± 0.44) were ranked in the order of the highest score in each program, in sequence (Table 3). The experts recommended that older adults complete a questionnaire in a simple language that they could easily understand and revise accordingly. It was recommended that the name of the content be modified so that older adults can understand it easily. Moreover, the size of the text and addition of picture letters were suggested so that older adults could accurately perceive the application content.

## 4. Discussion

This study developed a mobile application, “Smart Sleep”, to administer CBT to community-dwelling older adults with insomnia. The application was developed based on a systematic review, older adult preference survey, and expert evaluation. In this discussion of Smart Sleep applications, we discuss comparisons with existing literature, provide a deeper understanding of the development process, evaluate the strengths and limitations of the application, and detail the overall contribution of this study to the field.

### 4.1. Comparisons and Contributions to Literature

Among the previous experimental studies using CBT-I, in a comparative study between a group that used the application called the CBT-I Coach and a group that did not, the application improved the benefits of CBT-I. Sleep outcomes were reported to improve significantly [25]. Although previous experimental studies, especially those using the CBT-I Coach application [25], have shown improvements in sleep outcomes, this study is unique in that it specifically targeted community-dwelling older adults and provided motivational incentives. Subsequently, we are addressing a study on the self-management of insomnia in community-dwelling older adults using mobile applications reported in 2020 [30] and its similarities and differences with the current study. First, what they have in common is that the participants are older adults living in a community, and both studies provide empirical evidence that mobile CBT-I applications are available among older users. In addition, the arbitration and evaluation periods were the same: one week. However, while the current study developed a program that actively reflects the preferences and conveniences of experts and users at the development stage, this was not done in the 2020 study [30]. In contrast to this study, the 2020 study measured the quality of sleep specifically for elderly participants using the application. Therefore, a long-term follow-up study on the effects of the application developed in this study on sleep quality is required for comparison in these areas.

### 4.2. Reflections on Development Process

Sleep diaries are essential resources that provide useful evaluation information and guide the completion of CBT strategies [41]. Sleep activities and patterns were assessed using a sleep diary, and the appropriate sleep duration was fed back to the participants. It is an essential item in CBT-I and should be entered daily. Considering that the participants were older adults, the Smart Sleep application made it possible for sleep diary entries to be written in concise phrases, for easier understanding.

The sleep restriction method of the Korean version of the Clinical Guidelines for Insomnia was used to determine sleep time, and the target sleep and wake-up times were set while considering the patient’s sleep efficiency. These guidelines are as follows: gradually reduce or limit the total bedtime to increase the patient’s sleep efficiency; reduce the total bedtime by 15 or 30 min each week until reaching the target sleep efficiency of 85%; recalculate sleep efficiency in the next session; and evaluate the patient’s weekly symptoms such as fatigue, daytime sleepiness, vitality, and concentration. These guidelines are helpful in checking whether a patient’s subjective symptoms improve as sleep efficiency increases [42]. In the Smart Sleep application, as part of sleep stimulation and sleep restriction, participants were asked to input their sleep habits every Sunday for the week. Accordingly, the application checked the sleep habit status and adjusted the habits that stimulated sleep. In addition, the Sleep Habit Questionnaire includes information necessary for sleep hygiene. Through this overall management, managers can check real-time counseling and sleep habit questionnaires, thus improving their sleep habits.

### 4.3. Strengths and Limitations

The advantage of the Smart Sleep application is that community-dwelling older adults with insomnia, the application’s intended users, are motivated to receive feedback so that they can use the program consistently. In face-to-face CBT-I, patients write their sleep diaries on paper after waking and directly calculate the time spent in bed and total sleep time, which leads to poor adherence to treatment [43,44]. Additionally, ICT-based CBT-I requires greater program participation and better compliance [45]. Specifically, maintaining a sleep diary can burden the participants because it must be written daily. Accordingly, Smart Sleep created a “today’s to-do list” to increase users’ participation in the program, uploading today’s tasks on a daily basis so that the participants did not forget the task to be performed. The application also provided incentives after the tasks were completed. When older adults tapped on the main content, they were moved to the sub-content and could access it easily. Although the mobile application has advantages as an accessible and convenient instrument, it also has limitations in that it is difficult to control the environment and ensure compliance with the way the user conducts training, compared to face-to-face intervention [46]. This study has some limitations. It was difficult to compare and analyze the results of this study with those of existing studies owing to the lack of ICT-based CBT-I studies targeting older adults. Moreover, because of the small sample size and the study being limited to one area, the results cannot be considered representative of the entire older population. Hence, future research should confirm the effect of the intervention through a meta-analysis of new intervention studies that analyze the responses of a higher number of older participants.

### 4.4. Overall Contribution and Takeaways

The Smart Sleep application helped maintain users’ quality of sleep and improved their health through real-time exercise. The more the physical activity, the better the quality of sleep necessary to maintain health and ensure a good quality of sleep [18].

Although CBT applications for insomnia have been developed for adults, there is a lack of those that can be easily used by older adults. Smart Sleep applications can improve the sleep and quality of life of older adults by providing targeted solutions for insomnia. Unique features, such as motivation and improved usability, will enable the extended use of this tool. To address the inherent limitations of mobile device applications, such as environmental control issues, future research should be conducted on user experience, long-term compliance, and motivation-enhancement strategies for older adults. Other interventions and comparative studies with a broader participant base could provide valuable insights. Collectively, the “Smart Sleep” application is a step forward in responding to insomnia in older adults through innovative technology solutions. As research in this area progresses, further refinement and validation of these interventions will improve sleep quality and overall life satisfaction among older adults.

## 5. Conclusions

This study developed Smart Sleep, an application of CBT for community-dwelling older adults with insomnia, using a systematic literature review, preference analysis, and expert advice. The application provides sleep information, sleep-habit improvement programs, real-time counseling and exercise, sleep assistants, educational videos, relaxation training videos, and manager screens. The font was larger to make it easier for older adults to use the application, and pictograms were added. This application is expected to help older adults with insomnia by increasing their participation in CBT-I through feedback, incentives, monitoring, and alarm functions. Thus, there is a need to focus on the user experience of older adults, long-term implementation, and motivation-enhancing strategies. Furthermore, other studies and comparative studies on different populations should be conducted to compare the effects of “Smart Sleep” with those of existing approaches. Considering the environmental control challenges of this application, future studies should address research on user experience, long-term implementation, and strategies to enhance motivation among older adults.

## Figures and Tables

**Table 1:** Non-face-to-face service components for sleep-deprived and emotional older adults (preference) (*n* = 100).

Question Sections	Mean ^1^ (SD)	Min–Max
Listening to meditation music and sounds of nature	3.47 (1.25)	1–5
Reading a book	3.13 (1.32)	1–5
Stretching video before bed	3.09 (1.23)	1–5
Breathing exercises	3.01 (1.17)	1–5
Cognitive therapy through video	2.96 (1.18)	1–5
Knowing your sleep patterns	2.90 (1.29)	1–5
Providing feedback on sleep	2.81 (1.27)	1–5
Sleep hygiene education	2.75 (1.20)	1–5
Sleep stimulation control and sleep restriction therapy	2.73 (1.19)	1–5
Sleep diary	2.56 (1.10)	1–5

^1^ Preference points: 1 (not preferred at all) to 5 (extremely preferred).

**Table 2:** Usability assessment of experts.

Category	Component	Score (Mean ± SD)
Application quality	Mean score	4.58 ± 0.31
Engagement	Mean score	4.37 ± 0.44
	Entertainment	4.29 ± 0.48
	Interest	4.57 ± 0.53
	Customization	3.86 ± 0.37
	Interactivity	4.86 ± 0.37
	Target group	4.29 ± 0.48
Functionality	Mean score	4.35 ± 0.51
	Performance	4.42 ± 0.53
	Ease of use	4.00 ± 0.57
	Navigation	4.29 ± 0.48
	Gestural design	4.71 ± 0.48
Aesthetics	Mean score	5.00 ± 0.00
	Layout	5.00 ± 0.00
	Graphics	5.00 ± 0.00
	Visual appeal	5.00 ± 0.00
Information	Mean score	4.40 ± 0.37
	Accuracy of application description (in application store)	4.86 ± 0.37
	Goals	4.57 ± 0.53
	Quality of information	4.00 ± 0.82
	Quantity of information	5.00 ± 0.00
	Visual information	4.86 ± 0.37
	Credibility	5.00 ± 0.00
	Evidence base	2.57 ± 0.53
Application subjective quality	Mean score	4.78 ± 0.25
	Willingness to recommend application to others	4.43 ± 0.53
	Estimated number of uses per year	4.71 ± 0.48
	Willingness to pay for application	5.00 ± 0.00
	Overall star rating of application	5.00 ± 0.00
Total average		4.58 ± 0.31

**Table 3:** Usability testing by users’ assessment.

Category	Component	Score (Mean ± SD)
Application quality	Mean score	4.46 ± 0.42
Engagement	Mean score	4.26 ± 0.44
	Entertainment	4.10 ± 0.56
	Interest	4.20 ± 0.63
	Customization	4.00 ± 0.00
	Interactivity	4.40 ± 0.51
	Target group	4.60 ± 0.51
Functionality	Mean score	4.35 ± 0.36
	Performance	4.70 ± 0.48
	Ease of use	4.00 ± 0.00
	Navigation	4.40 ± 0.51
	Gestural design	4.30 ± 0.48
Aesthetics	Mean score	4.56 ± 0.50
	Layout	4.50 ± 0.52
	Graphics	4.70 ± 0.48
	Visual appeal	4.50 ± 0.52
Information	Mean score	4.72 ± 0.35
	Quality of information	4.40 ± 0.51
	Quantity of information	5.00 ± 0.00
	Visual information	4.80 ± 0.42
	Credibility	4.70 ± 0.48
Application subjective quality	Mean score	4.45 ± 0.45
	Willingness to recommend application to others	4.20 ± 0.42
	Estimated number of uses per year	4.20 ± 0.42
	Willingness to pay for application	4.70 ± 0.48
	Overall star rating of application	4.70 ± 0.48
Total average		4.46 ± 0.42

## Data Availability

The data presented in this study are available upon request from the corresponding author.
